# From One Domain to Another: The Pitfalls of Gender Recognition in Unseen Environments

**DOI:** 10.3390/s25134161

**Published:** 2025-07-04

**Authors:** Nzakiese Mbongo, Kailash A. Hambarde, Hugo Proença

**Affiliations:** 1 Department of Computer Science, University of Beira Interior, 6201-001 Covilhã, Portugal; n1947@ubi.pt (K.A.H.); hugomcp@ubi.pt (H.P.); 2 Department of Computer Science, Institute of Information and Communication Technologies, University of Luanda, Luanda P.O. Box 116, Angola; 3 IT: Instituto de Telecomunicações, University of Beira Interior, 6201-001 Covilhã, Portugal

**Keywords:** gender recognition, pedestrian attribute recognition, soft biometrics, model generalization, computer vision, deep learning

## Abstract

Gender recognition from pedestrian imagery is acknowledged by many as a quasi-solved problem, yet most existing approaches evaluate performance in a within-domain setting, i.e., when the test and training data, though disjoint, closely resemble each other. This work provides the first exhaustive cross-domain assessment of six architectures considered to represent the state of the art: ALM, VAC, Rethinking, LML, YinYang-Net, and MAMBA, across three widely known benchmarks: PA-100K, PETA, and RAP. All train/test combinations between datasets were evaluated, yielding 54 comparable experiments. The results revealed a performance split: median in-domain F1 approached 90% in most models, while the average drop under domain shift was up to 16.4 percentage points, with the most recent approaches degrading the most. The adaptive-masking ALM achieved an F1 above 80% in most transfer scenarios, particularly those involving high-resolution or pose-stable domains, highlighting the importance of strong inductive biases over architectural novelty alone. Further, to characterize robustness quantitatively, we introduced the *Unified Robustness Metric* (URM), which integrates the average cross-domain degradation performance into a single score. A qualitative saliency analysis also corroborated the numerical findings by exposing over-confidence and contextual bias in misclassifications. Overall, this study suggests that challenges in gender recognition are much more evident in cross-domain settings than under the commonly reported within-domain context. Finally, we formalize an open evaluation protocol that can serve as a baseline for future works of this kind.

## 1. Introduction

Gender recognition in pedestrian imagery is often treated as a solved problem. Benchmark accuracy is high, standard datasets are saturated, and new models routinely report incremental gains. Yet this confidence is an illusion. When tested outside the narrow confines of their training domain on different environments, sensors, or demographic distributions performance degrades catastrophically. The field has overestimated its progress and underestimated the fragility of its models [1].

This paper begins with a simple question: *Why does gender recognition fail across datasets?* We argue that the answer has been consistently misdiagnosed. First, deep models overfit to hidden dataset-specific signals, with spurious correlations rooted in clothing style, resolution, scene context, or demographic skew [2,3]. These cues are often imperceptible to human observers yet deeply embedded in the learned representations. Second, the dominant evaluation protocol for reporting dataset accuracy masks generalization failures. Training and testing on the same source inflates results and hides the brittleness of these systems under even minor distribution shifts [4,5].

Large scale pedestrian attribute datasets such as PETA [6], PA-100K [7], and RAP [8] offer enough diversity to support rigorous cross-domain testing. Yet most published models ignore this, evaluating only on the dataset they were trained on. Our experiments revealed that this practice leads to dangerously misleading conclusions. For example, a model trained on PETA and tested on RAP can perform worse than chance for some demographic subgroups, even when input resolution and label definitions are aligned. More complex architectures have not closed this gap. Deep residual networks [9], Swin Transformers [10], and recent sequence models like Mamba [11] achieve high accuracy on their source datasets, but degrade sharply under distribution shift. Architectural sophistication alone is not enough. Despite their capacity, these models remain brittle across domains. Prior work has focused on marginal architectural improvements—attention consistency [12], multi-scale localization [13], and loss function tweaks for imbalance mitigation [14]. But few have asked the more urgent question: *which design choices, if any, actually improve robustness across datasets?*

This work offers a diagnostic lens, not a new model. We conducted the first large-scale, cross-dataset evaluation of six gender recognition models. Across all nine train/test combinations of PA-100K, PETA, and RAP, we performed 54 experiments under a unified protocol. The results were unambiguous: models that performed well in-domain frequently collapsed under domain shift. Surprisingly, simpler models like ALM [13] sometimes transferred better than newer, more complex designs.

Our contributions are threefold:

- We introduce a unified cross-dataset evaluation protocol to benchmark robustness in gender recognition.
- We provide the first empirical comparison of six state-of-the-art models across all combinations of three major datasets, highlighting what transfers and what does not.
- We call for a shift in the field’s values: from chasing in-domain accuracy to prioritizing robustness as the core benchmark of progress.

The rest of this paper is organized as follows: Section 2 reviews prior work in pedestrian attribute recognition. Section 3 outlines our evaluation framework, datasets, and models, and introduces the Unified Robustness Metric (URM). Section 4 presents the quantitative and qualitative findings across 54 experiments. Section 5 analyzes systemic failure modes and reconsiders dominant modeling assumptions. Section 6 summarizes our findings and outlines the path forward for robust soft biometric evaluation.

## 2. Related Work

Pedestrian gender recognition is a foundational task in soft biometrics, with wide applications in surveillance, retail analytics, and demographic estimation. Progress in the field has largely been benchmark-driven, enabled by datasets like PETA [6], PA-100K [7], and RAP [8]. While these datasets vary in scene context and image diversity, they share a problematic trait: most models are trained and tested with the same dataset, creating brittle overfitting to domain-specific cues.

This overreliance on intra-dataset evaluation has been repeatedly critiqued. Jia et al. [4] and Gesnouin et al. [15] independently showed that models with high benchmark accuracy degrade sharply under distribution shift. Yet despite these warnings, most published methods continue to prioritize in-domain performance. Architectural innovations have attempted to localize attributes more precisely (e.g., ALM [13] via multi scale attention, VAC [12] via spatial consistency). Others have targeted label imbalance, such as LML [14], which reframes attribute learning as a label-balanced multi-label task. Hybrid approaches like YinYang-Net [16] fuse facial and body cues, while recent models like MAMBA [17] focus on sequence modeling and long-range dependency capture. Yet these models share a critical blind spot: they are rarely stress tested across datasets. When evaluated *out of domain* (OOM), architectural sophistication often fails to compensate for learned dataset priors. Worse, many models regress to near random prediction when tested on visually distinct domains, despite high in-domain F1-scores. Contextual factors such as occlusion, viewpoint, and resolution further compound these generalization failures [18]. Most architectures are not designed to disentangle signals from these confounders, and standard metrics fail to capture brittleness under domain shift.

In contrast to prior work, we conducted a rigorous cross-dataset evaluation of six representative models across three benchmarks, totaling 54 experiments. Unlike previous studies, we do not assume in-domain accuracy is a proxy for robustness. Instead, we introduce a unified robustness metric (URM) to quantify generalization explicitly. This shift in evaluation reveals uncomfortable truths about current model reliability and reframes the goal of soft biometric modeling as one of domain resilience, not benchmark dominance.

## 3. Methodology

### 3.1. Datasets and Preprocessing

We evaluated model performance using three standard pedestrian attribute recognition (PAR) datasets: PETA [6], PA-100K [7], and RAP [8]. These datasets offer complementary coverage across surveillance contexts, outdoor vs. indoor, frontal vs. occluded, and balanced vs. imbalanced, making them ideal for stress testing generalization.

PETA includes 19,000 images annotated with 61 soft biometric attributes, including gender, age, and clothing. Images are low resolution (ranging from 17×39 to 169×365 pixels), simulating long-range urban surveillance. PA-100K contains 100,000 images from 598 street level scenes. Each image is annotated with 26 attributes. The wide range of scenes and camera views makes PA-100K ideal for evaluating large-scale generalization under natural urban diversity. RAP offers 41,585 indoor surveillance images captured from 26 fixed cameras in a shopping mall environment. With 51 attributes and a high occlusion rate (∼33%), RAP introduces strong real-world complexity, especially in terms of partial visibility and multi-camera variation. To ensure consistency across datasets, we fixed the test set size at 1000 samples and split the remaining data into training and validation sets, as detailed in Table 1. These splits were held constant across all models evaluated.

**Figure 1:**
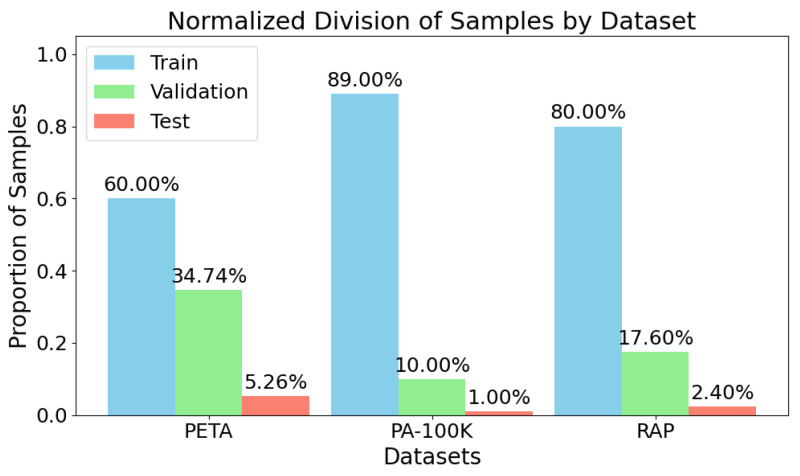
**Dataset sample distribution across splits.** Total number of images allocated to training, validation, and test sets for PETA, PA-100K, and RAP. All datasets used a consistent test size of 1000 images to ensure comparability.

Figure 1 visualizes the absolute distribution of samples across the three dataset splits. PA-100K has the largest training set, followed by RAP and then PETA.

#### Gender Label Distribution and Imbalance

Beyond dataset size and context, gender distribution plays a critical role in evaluating generalization, especially when models are trained in one domain and tested in another with a different label skew. Table 2 presents the absolute gender label counts, percentages, and imbalance levels for each dataset. Gender imbalance is quantified as the absolute percentage difference between male and female samples. RAP is heavily male-skewed (69.4%), while PETA is nearly balanced (50.3% male). PA-100K shows a moderate imbalance of 9.3%.

**Figure 2:**
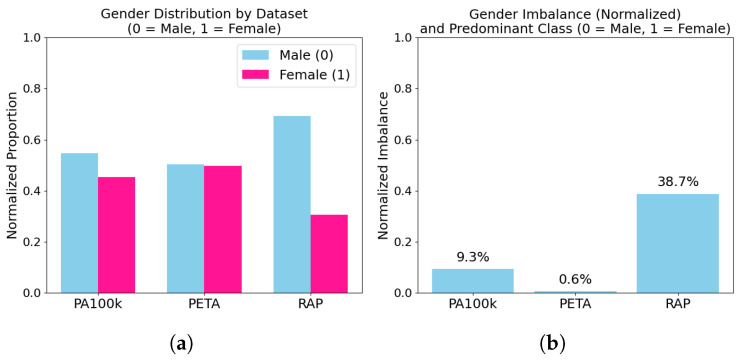
Gender distribution and imbalance visualization. (**a**): stacked bar plots showing the proportion of male vs. female samples in each dataset. (**b**): bar chart of absolute gender imbalance percentages. RAP exhibits strong male bias, while PETA is close to parity.

Figure 2 offers a visual breakdown of these statistics, showing both relative proportions (left) and imbalance magnitudes (right). RAP exhibits substantial gender skew, while PETA remains highly balanced—a critical factor in interpreting cross-dataset generalization results.

### 3.2. Model Selection

To evaluate generalization under domain shift, we benchmarked six state-of-the-art models originally designed for pedestrian attribute recognition (PAR). Five of these models perform multi-attribute prediction, while one (*YinYang-Net*) is explicitly tailored for gender classification. Despite differences in optimization, attention mechanisms, and task framing, the selected models represent the three dominant architectural paradigms in modern computer vision: convolutional backbones—ResNet-50, BN-Inception; transformer-based models—Swin Transformer Base; and more recent sequence modeling architectures—VisionMamba.

ResNet-50 [9] is a canonical convolutional network widely used for visual representation learning. BN-Inception [19] extends the Inception family [20] with batch normalization for improved multi-scale feature handling. Swin Transformer [10] uses hierarchical vision transformers with shifted windows to efficiently capture long-range dependencies. VisionMamba [11] replaces self-attention with state-space Mamba blocks for efficient sequential modeling in linear time.

Each model was initialized with ImageNet-pretrained weights and fine-tuned for binary gender classification. Multi-attribute models were reconfigured by replacing the output layer with a single sigmoid neuron predicting female class probability. Table 3 summarizes the six models, including their architectural backbone, publication year, and a brief description of their design focus. Together, they reflect a cross-section of methodological trends in the PAR literature from attention consistency (VAC) to label imbalance mitigation (LML) to recent Mamba-based sequence modeling (MAMBA).

Although the models evaluated in this study are not explicitly tailored for domain adaptation, they were deliberately selected among state-of-the-art Pedestrian Attribute Recognition (PAR) architectures to reflect current trends in the field. As future work, we plan to extend this analysis by integrating models specifically designed for domain adaptation, such as Domain-Adversarial Neural Networks (DANN) [21], which learn domain-invariant representations via adversarial training, and CORrelation ALignment (CORAL) [22], which efficiently aligns second-order statistics between source and target domains.

### 3.3. Evaluation Metrics

To assess model performance, we report standard classification metrics—Accuracy, Precision, Recall, F1-score, and AUC—computed over all nine train–test permutations of our cross-dataset evaluation protocol. While these metrics provide a baseline understanding of prediction quality, they are insufficient for evaluating cross-domain generalization, especially under dataset shift and class imbalance.

#### 3.3.1. Unified Robustness Metric (URM)

To directly quantify a model’s robustness across domains, we introduced the **Unified Robustness Metric (URM)**. URM consolidates two critical aspects of generalization:

1. **Cross-Domain Degradation (ACDD)**—the average relative drop in F1-score when models are tested out-of-distribution. 2. **Stability (SD)**—the standard deviation in performance across all domain pairs.

Let $D=\{D_{1},D_{2},D_{3}\}$ be the datasets (PETA, PA-100K, RAP), and let $F1_{i\to j}$ be the F1-score when trained on $D_{i}$ and evaluated on $D_{j}$. The metric is computed in four steps:

- **Cross-Domain Degradation (CDD)** for each i≠j:
  (1) $${\mathrm{CDD}}_{i\to j}=\frac{F1_{i\to i}-F1_{i\to j}}{F1_{i\to i}}$$
- **Average Degradation (ACDD)** across all six cross-domain pairs:
  (2) $$\mathrm{ACDD}=\frac{1}{6}\sum_{i\neq j}{\mathrm{CDD}}_{i\to j}$$
- **Stability (SD)** across all nine train–test F1 scores:
  (3) $$\mathrm{SD}=\sqrt{\frac{1}{9}\sum_{i,j}{\left(F1_{i\to j}-\bar{F1}\right)}^{2}}$$
- **Unified Robustness Metric (URM)**:
  (4) $$\mathrm{URM}=1-\left(\lambda \cdot \mathrm{ACDD}+\left(1-\lambda \right)\cdot \frac{\mathrm{SD}}{100}\right)$$

We set λ=0.7, giving a higher weight to degradation sensitivity. URM is bounded in [0,1], where higher values indicate stronger cross-domain generalization with stable performance.

#### 3.3.2. Interpretation

Unlike accuracy or F1, URM reflects both ***how far*** a model falls when transferred and ***how consistently*** it performs across shifts. A robust model should have low degradation and low volatility. URM rewards that combination.

### 3.4. Comparison with Existing Domain Robustness Metrics

Several well-established metrics exist for assessing domain shift and distribution alignment in machine learning, including Maximum Mean Discrepancy (MMD) [23], Correlation Alignment (CORAL) [22], Central Moment Discrepancy (CMD) [24], and the Wasserstein Distance. These metrics primarily operate in the feature space, quantifying how internal representations differ across domains based on statistical moments, distributions, or sample distances.

Our proposed Unified Robustness Metric (URM), in contrast, is designed to assess model performance from an output-centric perspective. Rather than requiring access to internal features or embedding spaces, URM relies solely on F1-scores across all train–test domain permutations. This makes it fully model-agnostic, easy to interpret, and applicable even in scenarios where internal representations are inaccessible (e.g., black-box systems).

Moreover, while metrics like MMD or CMD are valuable during domain adaptation or representation learning, URM complements them by quantifying final predictive stability and generalization gaps. Future work may include a hybrid evaluation that jointly considers distributional divergence and output-level robustness for a more holistic understanding of model reliability under domain shift, see Table 4.

### 3.5. Evaluation Workflow Overview

**Figure 3:**
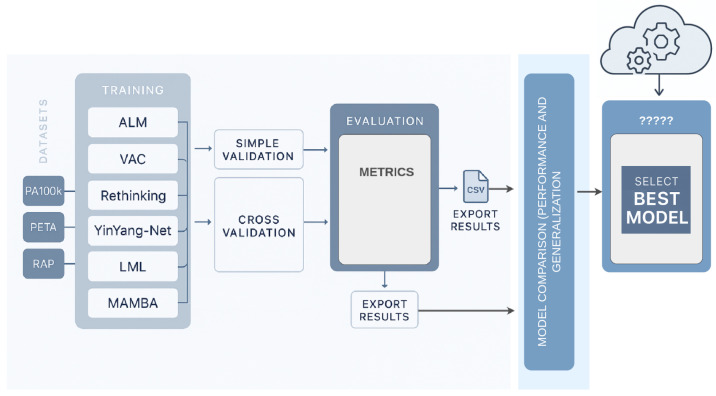
**Experimental workflow for gender recognition under domain shift.** Models were trained and evaluated across three datasets (PETA, PA-100K, and RAP), each characterized by different levels of visual complexity and label imbalance. The pipeline included intra- and cross-domain evaluations, yielding standard classification scores and robustness metrics (CDD, SD, and URM). This framework reveals not only how well models perform, but how reliably they generalize.

Figure 3 provides a visual summary of our evaluation pipeline. Models were trained and tested across three pedestrian attribute datasets—PETA, PA-100K, and RAP—with significant demographic, contextual, and visual variation. Predictions were analyzed using both traditional classification metrics and our domain-sensitive URM framework.

## 4. Experimental Results

We conducted a comprehensive evaluation of six state-of-the-art models (Rethinking, LML, MAMBA, ALM, YinYang-Net, and VAC) on binary gender recognition using the PA100k, PETA, and RAP datasets. Our objectives were twofold: (i) benchmark performance under standard intra-dataset training, and (ii) assess robustness under distribution shift via cross-dataset generalization.

### 4.1. Training Configuration

All models were implemented using the PyTorch framework and trained on two local GPUs: an NVIDIA RTX 4060 (8 GB) and a ZOTAC RTX 2080 (11 GB). The dataset splits defined in the PETA, PA-100K, and RAP benchmarks (NVIDIA Corporation, Santa Clara, CA, USA, ZOTAC Technology Limited, Fo Tan, New Territories, Hong Kong)—as presented in Table 1 in Section 3.1—were strictly preserved to ensure consistency across all experiments. Each model was trained independently for all nine train–test combinations defined by the cross-domain evaluation protocol, resulting in a total of 54 valid and systematically comparable configurations (6models×9scenarios). Fixed random seeds were used to guarantee reproducibility. On average, training each model per evaluation scenario required approximately 27 h, with total runs exceeding 100 to accommodate tuning and robustness checks. Input images were normalized and resized based on the input requirements of each model 224×224 for convolutional networks (e.g., ResNet-50) and 384×384 for transformer-based architectures (e.g., Swin Transformer). Additional training details, such as optimizer configuration, loss function, and early stopping strategy, are documented in Section 3 and the training hyperparameters used across all experiments are summarized in Table 5.

### 4.2. Quantitative Results

Table 6 reports the gender–attribute metrics (Acc, AUC, Prec, Rec, and F1) for the six state-of-the-art models across three popular pedestrian datasets: PA100k, PETA, and RAP. To make the discussion precise, we split the analysis into *intra-domain* (same train/test set) and *cross-domain* (different train/test sets) scenarios and conclude with the Unified Robustness Metric (URM, Table 7).

In Table 6, green underlines highlight the best values per metric (e.g., accuracy, AUC, precision, recall, and F1) within each model across all training–testing combinations. For instance, under the **Rethinking** architecture, training and evaluating on the same dataset (RAP→RAP) yielded the top performance across all metrics, including an AUC of 99.00. Interestingly, the same AUC value was observed when training and testing on PETA (PETA→PETA), although the remaining metrics were slightly lower.

#### 4.2.1. Intra-Domain Performance

All methods peaked when the evaluation domain matched the training domain. The best single score was obtained by **ALM**, which achieved an F1 of **99.54%** on RAP→→RAP. This large margin can be attributed to ALM’s adaptive local masking, which fit RAP’s high-resolution, pose-stable imagery and thereby suppressed background noise, while preserving fine gender cues. **YinYang-Net** (96.81%) and **VAC** (95.69%) followed closely; YinYang-Net benefited from its dual-branch silhouette pipeline, whereas VAC aggregated predictions from multiple weak learners, leading to balanced precision and recall. **LML** and **Rethinking**, both based on Swin-style patch encoders, still attained competitive F1 values between 89.29% and 95.96%, although the absence of explicit inter-patch regularization caused slight over-fitting to the richer RAP textures and PA100k. **MAMBA** achieved 92.50% F1 on RAP but remained behind the leaders because its deformable operators favored body deformation cues over global context.

#### 4.2.2. Cross-Domain Degradation

When the test distribution differed from the training set, the performance dropped by 18–60 percentage points, exposing three recurrent failure modes. First, a pose or viewpoint gap often confused silhouette-conditioned pipelines; for instance, YinYang-Net plunged from 96.81% to 57.09% F1 for the RAP→→PETA split, where viewpoints were more diverse. Second, resolution mismatch undermined feature localization: models trained on the high-resolution RAP images (164 × 384 px) struggled to detect small faces for PA100k (75 × 160 px), and ALM, for example, lost 18.41 percentage points in precision on RAP→→PA100k and 31.74 percentage points in precision on RAP→→ PETA. Third, label imbalance shifted decision thresholds; the 11:9 male–female ratio in PA100k contrasted with the balanced 5:5 ratio in PETA, which reduced LML’s recall to 67.02% on PA100k→→PETA.

#### 4.2.3. Model-Specific Robustness

**VAC** exhibited the smallest average degradation (ACDD 0.1078) and the highest URM (0.8986). Its patch-level voting smoothed decision boundaries and even led to strong cross-domain generalization. **LML** and **Rethinking** achieved similar URM values (0.8709 and 0.8594, respectively); their large receptive fields handled moderate domain shift, but the absence of explicit alignment kept the degradation around 0.15–0.16. **ALM** preserved an exceptionally high precision within domain, yet proved brittle under distribution change, as reflected by a standard deviation of 10.03 and a URM of 0.8587. **MAMBA** (URM 0.844) suffered from style sensitivity: its deformable attention tended to latch onto color palettes, which varied greatly across datasets. Finally, **YinYang-Net** obtained the lowest URM (0.7797), because its reliance on precise silhouettes made it highly vulnerable to unseen poses and annotation styles; both the highest average degradation (0.2546) and the greatest variability (SD 14.02) confirmed this weakness.

#### 4.2.4. Unified Robustness Metric

The URM synthesizes average degradation and variability into a single figure of merit. Although ALM recorded the top closed-set F1, VAC is the most reliable choice for deployment, as it only sacrificed a few intra-domain points to gain a markedly stronger generalization. By contrast, YinYang-Net’s high closed-set accuracy hides a pronounced sensitivity to distribution shift.

### 4.3. Qualitative Analysis

**Figure 4:**
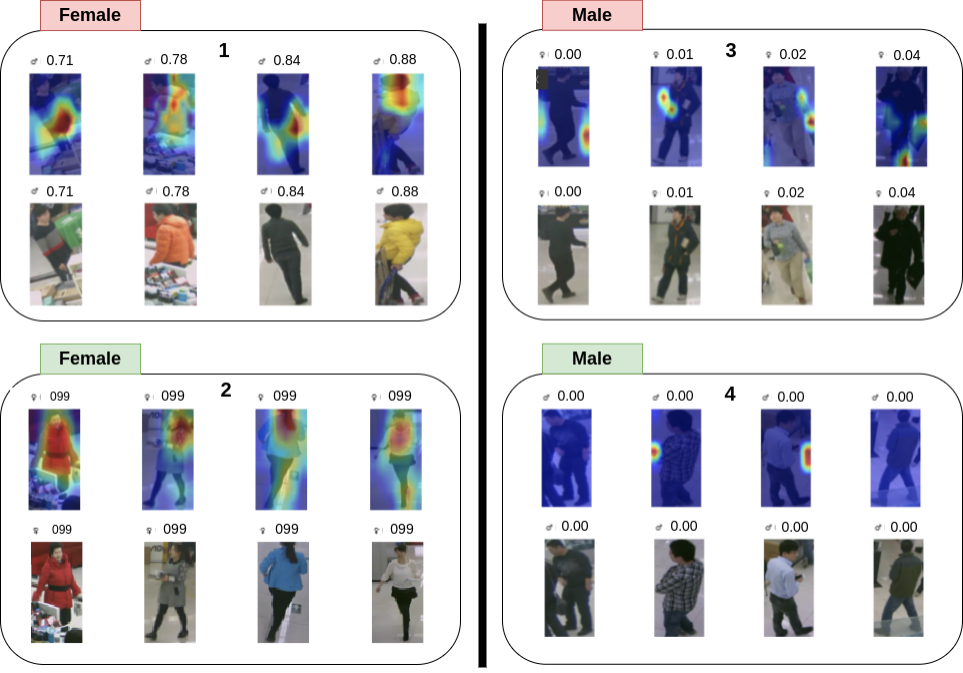
In Figure 4, the green and red colors represent the model’s prediction outcomes. The areas highlighted in green indicate successful cases where the model made confident and correct predictions. In contrast, the red areas correspond to failure cases, where the model’s predictions were incorrect.

Beyond the numerical scores reported in Section 4.2, a visual inspection of model behavior clarified *why* certain errors arose and which architectural choices promoted—or undermined—generalization. To that end, we examined class–posterior scores and Grad-CAM saliency maps [26] for a curated subset of images spanning the three datasets. Figure 4 juxtaposes representative successes (left) and failures (right) for each model.

**Confidence profiles.** Correct predictions were typically accompanied by high posterior probabilities (≥0.70); the distribution of those scores was narrow, indicating unequivocal evidence in the input. Misclassifications, in contrast, rarely presented borderline scores: the models were *over-confident* even when they erred, with several female images mislabeled as male at scores of 0.95–0.99. Such behavior reveals a calibration problem that cannot be detected from accuracy or F1 alone.

**Saliency patterns.** Across all architectures, the salient regions for male subjects concentrated on the head–torso silhouette, as well as posture cues. Female subjects were likewise identified through body outline, yet an additional reliance on hair length and apparel was evident, especially in LML and YinYang-Net. Images containing short-haired women, neutral or traditionally ’masculine’ clothing, or low-key illumination mislead the models; Grad-CAM maps show the attention shifting from the face to the outline of backpacks or broad hand gestures, confirming a bias towards accessory context rather than inherent morphological traits.

**Dataset shift and robustness.** The activation maps exposed the extent to which each model refocused under domain shift. ALM preserved its attention on the torso–head region, even when the resolution dropped, which partly explains its superior in-domain precision. ALM preserved its attention on the torso–head region, even when the resolution dropped, which partly explains its superior in-domain precision and comparatively robust cross-domain performance. VAC distributed attention across broader regions, often spanning the upper body; such redundancy mitigated the effects of noisy inputs and supported the model’s strong in-domain and cross-domain generalization. Conversely, YinYang-Net relied heavily on silhouette segmentation and therefore failed when poses deviated from the canonical upright stance so common in RAP. These observations are consistent with the degradation figures in Table 7, reinforcing the link between saliency stability and empirical robustness.

**Implications.** This qualitative evidence underscores two critical limitations: first, an over-confidence in the presence of gender-ambiguous cues, and second, an over-reliance on accessories that correlate spuriously with gender. Addressing the former calls for calibration techniques or cost-sensitive training, whereas the latter motivates stronger data augmentation or explicit debiasing of contextual attributes. ALM’s comparatively stable saliency suggests that adaptive local masking is a promising direction for mitigating such biases, yet its attention still drifts under extreme resolution changes, signaling room for improvement.

## 5. Discussion

The empirical evidence collected in Section 4.2 and Section 4.3 converges on a single message: high in-domain accuracy is an unreliable predictor of real-world reliability. Although every model achieved at least 90% F1 when evaluated on the dataset it was trained on, performance collapsed—sometimes to chance level—once the test distribution diverged. This section interprets those results, relates them to architectural design choices and dataset characteristics, and outlines directions for future research.

### 5.1. Why In-Domain Champions Stumble out of Domain

Table 6 shows that **ALM** reached a near-perfect F1 of 99.54% on RAP→RAP, yet Table 7 ranks the same model fourth for the Unified Robustness Metric (URM). The discrepancy is explained by a sharp precision drop whenever the input resolution or illumination profile deviated from that of RAP. In contrast, **VAC** never topped the leaderboard for closed-set accuracy, but its attention-consistency constraint regularized the feature extractor sufficiently to keep the average cross-domain degradation below 14%. Thus, robustness stemmed less from headline accuracy than from inductive biases that retained saliency under visual perturbations.

### 5.2. The Architectural Factor

The qualitative study in Figure 4 reveals that convolutional backbones with explicit locality constraints—BN-Inception for ALM and ResNet-50 for VAC—focused stably on the torso–head region, whereas the transformer-based LML and Rethinking occasionally shifted attention to contextual artifacts such as backpacks. This finding nuances the common assumption that larger receptive fields automatically improve generalization. Equally instructive is the behavior of **YinYang-Net**: its silhouette–centric design excelled when body contours were clean but failed catastrophically on oblique or occluded views, producing the lowest URM in Table 7. The newest architecture, **MAMBA**, suffered from style sensitivity; its state-space blocks captured long-range dependencies, yet also amplified correlations with background color palettes, leading to unstable cross-domain scores.

### 5.3. The Dataset Factor

**Figure 5:**
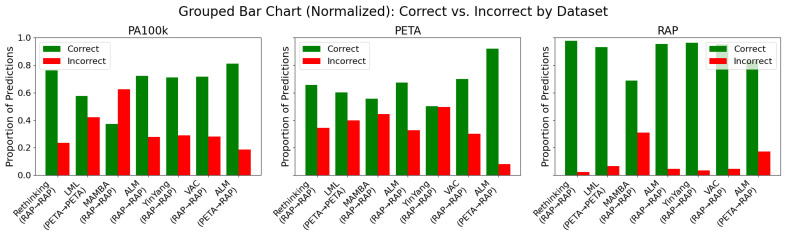
This figure is organized by dataset (PA100k, PETA, and RAP), providing a clear view of each model’s performance across different domains. Green bars represent correct classifications, while red bars indicate errors. Each subplot groups models based on their proportions of correct and incorrect predictions during testing, facilitating both intra- and cross-domain analysis. Notably, the RAP dataset demonstrated greater overall robustness, with the ALM model achieving the lowest error rate. In contrast, the PA100k and PETA datasets exhibited higher performance variability and error rates, highlighting the generalization challenges posed by domain shift.

Performance disparities are not dictated by architecture alone. As illustrated in Figure 5, RAP was consistently the *easiest* domain: even MAMBA surpassed 90% F1 when both training and testing on RAP, whereas the same model barely broke 65% when transferred to PETA (Table 6). This difference originates from RAP’s controlled viewpoints and higher resolution, attributes that simplify silhouette extraction and facial cue detection. Conversely, PA-100K’s low resolution and the near gender parity of PETA exposed biases that remained hidden in male-dominated RAP. Table 2 quantifies this skew; any model calibrated on a 7:3 male–female ratio will inevitably mis-score female samples when moved to a balanced test set.

### 5.4. Calibration and Over-Confidence

**Figure 6:**
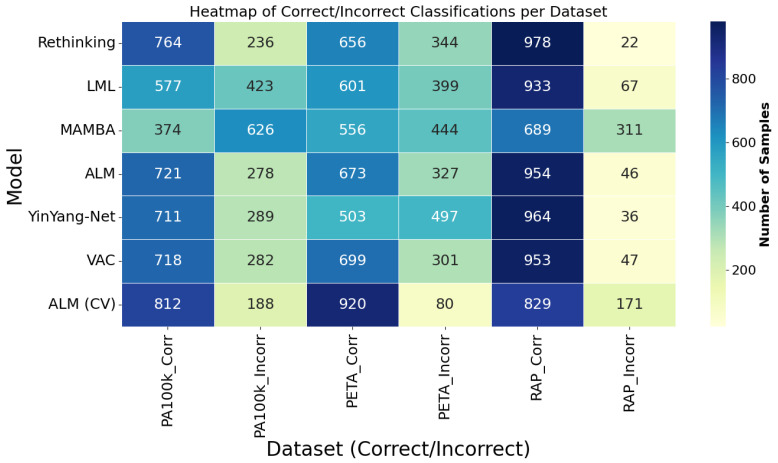
This figure presents a heatmap that provides a consolidated view focused on the models. Each row corresponds to a model, and the columns indicate the number of correct and incorrect predictions in each dataset. The color intensity allows for quick identification of models that stood out in performance. The ALM model, especially in the cross-validation (CV) version, stood out with the highest values of correct classifications and the lowest error rates, particularly on the PA100k and PETA datasets. This representation is especially useful for recognizing performance patterns and assessing the consistency of each model across different domains.

A less visible but equally consequential problem is miscalibration. Figure 6 illustrates repeated cases in which female pedestrians were assigned male probabilities exceeding 0.95. Such over-confidence is worrisome for downstream systems that rely on confidence thresholds to trigger actions or audits. Neither F1 nor AUC captures this pathology, underscoring the need for evaluation protocols that include reliability diagrams or expected calibration errors.

### 5.5. Implications for Practice

Taken together, these observations recommend three practical guidelines. First, any deployment pipeline should incorporate explicit cross-domain validation; otherwise, the risk of demographic harm or operational failure remains opaque. Second, architectural novelty should be judged not only by in-domain gains but by the stability of saliency and class posterior distributions under shift. Third, dataset design must prioritize diversity in resolution, pose, and demographic balance; benchmarks that fail to stress those axes inadvertently reward overfitting.

### 5.6. Future Work

This study highlights two key methodological directions for future research. First, *domain-centric training*—curriculum learning or meta-learning strategies that alternate between source domains—could reduce dependence on dataset-specific biases and improve generalization. Second, *calibrated inference* methods such as post hoc temperature scaling or Bayesian ensembling may help mitigate over-confidence and produce more trustworthy predictions under distribution shift.

Additionally, future work should include *demographic bias analysis*, assessing model performance across subgroups such as gender, age, ethnicity, and clothing style. This step is critical to ensure algorithmic fairness and ethical deployment in real-world applications.

It is also important to consider the role of the datasets themselves. The RAP dataset appeared particularly favorable for most models, with high accuracy rates consistently observed across the board. This may reflect the dataset’s visual clarity or the richness of its gender representation.

## 6. Conclusions

This study presented a thorough cross-dataset comparative evaluation of six publicly available models that we considered to represent the state-of-the-art in gender recognition based on pedestrian images. By systematically testing these models across three widely known datasets (PA100k, PETA, and RAP), we revealed important insights about their generalization abilities beyond isolated training scenarios. Although most models performed well when evaluated on the same dataset used for training, their robustness notably declined when exposed to different domains. Among our experiments, the ALM model yielded the most remarkable consistency, standing out for its ability to maintain relatively high F1-scores in cross-dataset settings. This highlights its potential for real-world applications where variability in data is the norm. In contrast, other models such as MAMBA struggled to effectively adapt to unseen domains, despite their modern architectural frameworks. Complementary, our qualitative analyses further illustrated ALM’s resilience under visually challenging conditions, such as occlusions or lighting inconsistencies. These findings collectively reinforce the value of cross-dataset evaluation as a critical benchmark for advancing soft biometric recognition. Moving forward, the development of gender recognition systems must prioritize not only performance in relatively similar learning/test settings (i.e., within domain) but particularly should evaluate adaptability, stability, and generalization across diverse operational environments, which will play a role in the deployment of these kinds of systems in real-world scenarios.

## Figures and Tables

**Table 1:** **Dataset partitioning.** Division of the PETA, PA-100K, and RAP datasets into training, validation, and test subsets. Each dataset used a fixed test size of 1000 images to maintain evaluation consistency.

Dataset	Training	Validation	Test	Total Samples
PETA	11,400	6600	1000	19,000
PA-100K	89,000	10,000	1000	100,000
RAP	33,268	7317	1000	41,585

**Table 2:** **Gender distribution across datasets.** Absolute counts, class percentages, and gender imbalance scores for male and female categories in PETA, PA-100K, and RAP. Imbalance was calculated as the absolute difference between male and female proportions.

Dataset	Male	Female	Total	Male	Female	Imbalance
PA-100K	54,664	45,336	100,000	54.66	45.34	9.32
PETA	9557	9443	19,000	50.30	49.70	0.60
RAP	28,759	12,701	41,460	69.37	30.63	38.74

**Table 3:** **Overview of evaluated models.** Each model was benchmarked for binary gender classification. The table lists the base architecture, publication year, and key methodological contribution as described in the original source.

Model	Backbone	Year	Key Contribution
ALM [13]	BN-Inception	2019	Weakly supervised multi-scale attention maps for improved attribute localization.
VAC [12]	ResNet-50	2019	Enforces spatial attention consistency to stabilize predictions under geometric transformations.
Rethinking [4]	Swin Transformer	2021	Revisits label-dependency structures in multi-attribute prediction.
YinYang-Net [16]	ResNet-50	2022	Combines face and body cues via matrix-based attention fusion for gender recognition.
LML [14]	Swin Transformer	2024	Proposes label-balanced loss to mitigate prediction skew in multi-label classification.
MAMBA [17]	VisionMamba	2024	Introduces Mamba blocks for efficient modeling of long-range dependencies in attribute recognition.

**Table 4:** Comparison of domain robustness and divergence metrics.

Metric	Requires Access to Embeddings	Ease of Interpretation	Level of Analysis
**MMD** [23]	Yes (✓)	Moderate—requires understanding of RKHS and kernel embeddings	Feature space (mean discrepancy in high-dimensional space)
**CORAL** [22]	Yes (✓)	Moderate/High—based on covariance matrix alignment	Feature space (second-order statistics)
**CMD** [24]	Yes (✓)	Moderate—depends on interpretation of high-order moments	Feature space (moments up to order 5)
**Wasserstein Distance** [25]	Yes (✓)	Low—based on optimal transport theory; computationally complex	Probability distribution space (transport-based)
**URM (Ours)**	No (✗)	High—directly based on F1-score across domains	Model output level (cross-domain predictive stability)

✓ Yes/✗ No; ease of interpretation: High, Moderate, Low. URM = Unified Robustness Metric proposed in this study.

**Table 5:** Training hyperparameters used across all experiments.

Hyperparameter	Value/Setting
Optimizer	Adam (lr = 0.0001)
Batch size	32
Epochs	60
Early stopping	Patience = 10 epochs
Learning rate schedule	ReduceLROnPlateau (patience = 5, factor = 0.1)
Weight Decay	0.0005
Data augmentation	AutoAugment (p = 0.5), Resize, RandomHorizontalFlip,
	Normalize

**Table 6:** Performance of the different methods for the gender attribute across the PA100k, PETA, and RAP datasets.

Train	Val	Acc	Auc	Prec	Rec	F1
PA	PA	92.19	97.00	90.00	88.93	89.46
PA	PE	79.02	89.00	76.00	67.10	71.27
PA	RA	89.00	96.00	86.00	82.00	83.95
PE	PA	81.00	89.00	77.00	89.00	82.57
PE	PE	93.21	99.00	94.00	94.07	94.03
PE	RAP	79.00	91.00	72.24	65.10	68.48
RA	PA	79.80	90.00	70.00	91.00	79.13
RA	PE	74.90	85.00	75.00	90.50	82.02
RA	RA	96.12	99.00	96.00	95.92	**95.96**
(a) Rethinking						
**Train**	**Val**	**Acc**	**Auc**	**Prec**	**Rec**	**F1**
PA	PA	90.00	96.00	87.83	88.00	87.91
PA	PE	76.81	86.00	73.43	67.02	70.08
PA	RA	86.00	94.00	80.02	81.80	80.90
PE	PA	80.28	88.00	75.89	75.30	75.59
PE	PE	90.97	97.00	90.00	89.19	**89.59**
PE	RA	82.40	90.00	73.75	81.19	77.29
RA	PA	74.50	82.00	69.60	80.00	74.44
RA	PE	68.00	76.00	68.50	78.08	72.98
RA	RA	92.20	97.00	88.50	90.10	89.29
(b) LML						
**Train**	**Val**	**Acc**	**Auc**	**Prec**	**Rec**	**F1**
PA	PA	79.80	87.00	75.00	78.00	76.47
PA	PE	65.60	71.00	64.50	68.30	66.35
PA	RA	75.00	84.00	64.80	80.20	71.68
PE	PA	67.00	73.00	68.03	70.00	69.00
PE	PE	90.10	96.00	88.00	92.30	90.01
PE	RA	68.00	74.00	75.06	65.00	70.06
RA	PA	63.30	70.00	61.00	70.00	65.19
RA	PE	65.30	76.00	69.00	91.00	78.49
RA	RA	91.50	97.00	88.00	97.51	**92.50**
(c) MAMBA						
**Train**	**Val**	**Acc**	**Auc**	**Prec**	**Rec**	**F1**
PA	PA	90.50	96.00	81.82	89.58	85.52
PA	PE	76.66	85.00	75.00	71.63	73.28
PA	RA	87.99	94.00	84.62	79.74	82.11
PE	PA	77.75	84.00	72.73	62.54	67.25
PE	PE	93.30	98.00	100.00	95.24	97.56
PE	RA	83.18	89.00	92.21	86.07	89.02
RA	PA	74.02	86.00	81.14	89.74	85.22
RA	PE	74.18	86.00	67.95	88.35	76.82
RA	RA	96.00	99.00	99.69	99.42	**99.54**
(d) ALM						
**Train**	**Val**	**Acc**	**Auc**	**Prec**	**Rec**	**F1**
PA	PA	90.58	96.00	87.47	88.50	87.98
PA	PE	63.05	68.00	63.05	62.90	62.97
PA	RA	88.10	94.00	87.96	88.10	88.03
PE	PA	61.69	60.00	63.58	61.69	62.62
PE	PE	90.88	96.00	90.85	92.89	91.86
PE	RA	69.48	61.00	64.50	69.48	66.90
RA	PA	70.44	83.00	76.18	70.44	73.20
RA	PE	49.82	68.00	68.73	48.82	57.09
RA	RA	96.81	99.00	96.82	96.81	**96.81**
(e) YinYang-Net						
**Train**	**Val**	**Acc**	**Auc**	**Prec**	**Rec**	**F1**
PA	PA	87.89	94.00	94.00	84.59	89.05
PA	PE	76.72	86.00	81.23	62.78	70.82
PA	RA	88.93	94.00	88.92	88.93	88.92
PE	PA	75.86	84.00	79.27	64.43	71.08
PE	PE	79.96	92.00	79.23	75.50	77.32
PE	RA	84.18	91.00	84.47	84.18	84.32
RA	PA	76.00	83.00	76.86	76.09	76.46
RA	PE	69.89	83.00	73.86	69.89	71.82
RA	RA	95.69	99.00	95.69	95.69	**95.69**
(f) VAC						

**Table 7:** Unified Robustness Metric (URM) results for each evaluated model. **VAC** achieved the highest URM score, indicating the strongest generalization under domain shift, while **YinYang-Net** showed the lowest robustness. ACDD: average cross-domain degradation. SD: standard deviation of F1-scores. URM: unified robustness metric (higher is better).

Model	ACDD	SD (F1)	URM
**VAC**	0.1078	8.65	**0.8986**
LML	0.1542	7.04	0.8709
Rethinking	0.1632	8.8	0.8594
ALM	0.1589	10.03	0.8587
MAMBA	0.1828	9.34	0.844
**YinYang-Net**	0.2546	14.02	**0.7797**

## Data Availability

The datasets analyzed during the current study are publicly available: PA-100K: https://github.com/xh-liu/HydraPlus-Net (accessed on 1 July 2025); PETA: https://mmlab.ie.cuhk.edu.hk/projects/PETA.html (accessed on 1 July 2025); RAP: https://www.rapdataset.com/ (accessed on 1 July 2025).
